# Mr. Bartley in Reply to Mr. Fogo

**Published:** 1804-05-01

**Authors:** O. W. Bartley

**Affiliations:** Nailsworth, Gloucestershire


### *To the Editors of the Medical and Physical Journal.*

GENTLEMEN,

THE reason of my now troubling you, is to remark some critical observations of Mr. Fogo, in your Journal, No. 59, p. 67, on a paper of mine concerning the delivery of the placenta, which you have done me the honour to insert in a former number.

In the first place, he seems dissatisfied with the application tion of the term morbid. I cannot, I confess, agree with him as to its impropriety, notwithstanding all the arguments he has advanced on the subject. I conceive morbid and diseased to be synonimous terms. Mr. F. supposes it probable that "the placenta remained partly attached to the uterus from its *atonic state*." He will not surely contend that atony is not a disease. Every circumstance occurring contrary to the usual economy of Nature, so as to derange the animal system, may be justly, I think, called *morbid* or diseased. The retention of the placenta, arising from an "atonic state of the uterus," is not certainly to be considered as a *natural* circumstance, and might therefore with propriety be termed morbid. As it is, however, of little use to cavil about words, I shall proceed to consider the more important parts of the case.

Mr. F. is of opinion, that hæmorrhage still continued to exist during the four hours the placenta was retained, and that I gained no advantage by the delay, as the patient *could not* increase in strength. Both these positions I positively deny. The placenta adhered to the fundus uteri, and did not "block up the vagina." If hæmorrhage did exist, there was sufficient room for the blood to have escaped in a fluid state, and consequently it would have appeared externally. The clots I removed did not, as Mr. F. has conjectured, nearly amount in weight to "two pounds or one." It did not exceed what might be expected to have coagulated (during the hæmorrhage, which succeeded the birth of the child) from the recumbent posture of the patient, and was afterwards propelled into the vagina by slight contraction of the uterus.

As to the strength of my patient, it evidently increased to a very considerable degree; during the time I waited, she had recovered in a great measure from that debility I observed on my first arrival, before I attempted to remove the placenta; her pulse, at first excessively weak, was become tolerably strong and regular; her spirits repaired, and her countenance no longer exhibited that cadaverous appearance I described. I may therefore, I think, fairly presume from these circumstances, that no hæmorrhage did then exist. Her importunities for the removal of the placenta (to which Mr. F. attributes her salvation) were the effects of weariness from remaining so long on the bed in an uneasy posture, and a consequent wish of being changed and placed in a more comfortable situation. The subsequent faintings were, I conceive, occasioned partly by the pain felt from the introduction of my hand into the uterus, which had somewhat receded and contracted, and partly by her dread of the operation.

"I

"I also (as Mr. F. expresses himself) am one of those who can see no safety in allowing the placenta to remain four hours, (or one) in case of an existing though not apparent hæmorrhage." If I had the slightest cause to suspect its existence, I should remove every thing which would impede the contraction of the uterus without hesitation, and whether I suspected it or not, I should not, as Mr. F. *pleasantly* supposes, "sit pulling the cord," thereby increasing or perhaps causing the evil I wish to avoid. In the case in question, I gently strained the funis five or six times in the course of the four hours (for about so often the pains occurred) in order to ascertain whether contraction (indicated by pain) had at all furthered the expulsion of the placenta. Mr. F. may probably be a greater adept at pulling a cord than myself, for he seems to have rung round the changes merrily on me and Mr. Marson; I leave others to determine with how much judgment.

I shall only add, that the arguments of Mr. F. are not at all calculated to convince me of any impropriety of conduct in this particular, and that if a similar case should occur in my practice, attended with like circumstances, I should, notwithstanding the plausibility of his reasoning, act in just the same manner without dreading the result.

I am, &c.

*Nailsworth, Gloucestershire,*
*April* 10, 1804.

O. W. BARTLEY.

